# Editorial: Alzheimer's disease: new insights into biomechanisms and therapeutic target

**DOI:** 10.3389/fnagi.2025.1569412

**Published:** 2025-03-05

**Authors:** Yang Zhang, Jie Zhu, Maoli Duan

**Affiliations:** ^1^Department of Neurology, Affiliated Drum Tower Hospital of Nanjing University Medical School, Nanjing, China; ^2^Department of Neurology, Neuroscience Center, The First Hospital of Jilin University, Changchun, China; ^3^Division of Neurogeriatrics, Department of Neurobiology, Care Sciences and Society, Karolinska University Hospital, Karolinska Institute, Stockholm, Sweden; ^4^Department of Otolaryngology Head and Neck Surgery and Audiology and Neurotology, Karolinska University Hospital, Karolinska Institute, Stockholm, Sweden; ^5^Division of ENT Section, Department of Clinical Science, Intervention and Technology, Karolinska Institute, Stockholm, Sweden

**Keywords:** Alzheimer's disease, amyloid-beta, therapeutic targets, sphingolipid metabolism, clathrin-mediated endocytosis

Alzheimer's disease (AD) is the most common age-related dementia and primarily characterized by the extracellular deposition of amyloid-beta (Aβ) plaques and intracellular neurofibrillary tangles in the brain. Over the last decade, significant progress has been made in understanding etiology and therapeutic targets of AD. However, due to the complex pathogenesis of AD, we still face severe challenges in clinical treatment.

The Research Topic of this issue is “*Alzheimer's disease: new insights into biomechanisms and therapeutic targets*”, which aims to explore the various molecular mechanisms of AD pathogenesis and discover new promising potential therapeutic targets. Here are 10 articles, including 6 original works and 4 reviews, which collectively address various facets of biomechanisms aspects of biological mechanisms and therapeutic targets in the context of AD, such as abnormal sphingolipid metabolism, clathrin-mediated endocytosis (CME) and the role of hypoxia in the pathogenesis of AD. The contents of these articles are briefly summarized as follows:

1. This study explored changes in the effective connectivity (EC) network of the basal forebrain in AD patients before and after donepezil intervention using rs-fMRI data and the Granger causality analysis (GCA) approach. The findings suggested that donepezil enhanced the strength of connections between the basal forebrain with the default mode network and middle occipital gyrus, thereby improving cognitive function in AD patients. These results are helpful for better understanding of the neural mechanism of donepezil in the treatment of AD and for finding clinical targets for intervention (Yang et al.).

2. The role of sphingolipids was investigated in AD brains, Cerad score B brains and induced pluripotent stem (iPS) cells. AD brains exhibited higher levels of sphingosine (Sph), total ceramide 1-phosphate (Cer1P) and total ceramide (Cer), while higher levels of sphingomyelin (SM) exclusively in Cerad-B brains, which suggested the importance of sphingolipid metabolism in AD pathology (Uranbileg et al.).

3. The neuroprotective effects and mechanism of 4,4′-methylenediphenol in AD model worms were examined in this study. The results showed that 4,4′-methylenediphenol improved motility and stress tolerance, while also delaying the onset of paralysis and senescence in the AD model. Along with upregulating the expression of SKN-1, SOD-3, and GST-4 in the corresponding GFP reporter lines and promoting the nuclear translocation of DAF-16, 4,4′-methylenediphenol also increased antioxidant activity and decreased Aβ toxicity, suggesting to further study the anti-AD effects of Gastrodia elata and its active ingredients (Yu et al.).

4. The study investigated the degree of GluN2A or GluN2B-containing NMDAR contribute to Aβ (1–42) mediated impairments of hippocampal function. The results showed that GluN2A subunit knockdown modified the membrane characteristics of hippocampal neurons and lowered the amount of long-term potentiation (LTP). LTP's early phase was diminished by GluN2B knockdown, while its later stages were unaffected. However, neither GluN2A nor GluN2B-containing NMDAR mediated the aged hippocampus's susceptibility to Aβ-mediated impairments of LTP. It suggested that the pathogenic effects of oligomeric Aβ (1–42) on hippocampal function were not propagated via NMDAR in the aged hippocampus (Südkamp et al.).

5. Abnormal dynamic functional connectivity (DFC) is a neuroimage feature of AD. Energy landscape analysis was applied to the resting-state fMRI data to characterize the aberrant brain network dynamics in AD patients and controls. Their results suggested that the co-activation state could be important to cognitive processing and AD group possibly raised cognitive ability by increasing the occurrence and transition between the impaired cognitive control and sensory integration states (Xing et al.).

6. The authors carried out a MR study to investigate causal links between a variety of immune cell phenotypes and AD using GWAS data from European cohorts. Findings showed that HLA DR expression on B cells and the absolute number of CD28–CD4–CD8—T cells were associated with a protective effect against AD, while 13 other immunological phenotypes were risk factors. This work offers novel insights into the immunopathogenesis of AD (Zhang et al.).

7. This review summarized the research progress on cognition-related neural network oscillations, and complex anatomical and projective relationships between nucleus basalis of Meynert (NBM) and other cognitive structures. The important functions of the NBM in neuromodulation were also reviewed. The authors believe that neuromodulation based on the NBM plays an important and complex role in treating neurodegenerative disorders (Jiao et al.).

8. The review explored the impact of clathrin-mediated endocytosis (CME) on AD etiology. Disrupted CME in neurons leads to synaptic dysfunction, Aβ processing, and Tau pathology in early AD pathogenesis. CME alterations also affect the ability of astrocytes and microglia to clear Aβ, and neuroinflammation. Dysregulated CME in these cells highlights its AD pathophysiological implications (Jaye et al.).

9. Hypoxia has long been identified as one of the potential causes of AD. This review elucidated the effect of hypoxia-inducible factors-1α and oxidative stress in AD process, including inflammation, Aβ deposition, and mitochondrial dysfunction. The authors speculated that antioxidants could be a potential therapeutic approach for AD (Tao et al.).

10. This systematic review seeked to explore the correlation between sTREM2 levels and AD progression through a meta-analysis of sTREM2 levels in both cerebrospinal fluid (CSF) and blood. The findings indicated a positive correlation between elevated CSF sTREM2 levels and a higher risk of AD and MCI. Besides, plasma sTREM2 levels were notably higher in the AD group, which may serve as a promising biomarker for AD (Wang et al.).

In short, all articles published in this Research Topic contribute to a better understanding of the biomechanisms underlying AD. The findings have the potential to affect clinical practice, inspire new therapeutic targets, and guide the development of novel intervention and approaches of AD. We look forward to additional revolutionary studies in this Research Topic that provides a new perspective.

